# Supplementary material for: Bayesian Estimation of Mosaic Loss of Chromosome Y from Bulk RNA Sequencing Data
Source: bioRxiv. 2026 May 23:2026.05.20.726153. Preprint. [Version 1] doi: 10.64898/2026.05.20.726153 (PMC13228575; doi:10.64898/2026.05.20.726153)
Supplement: Supplement 2 [file NIHPP2026.05.20.726153v1-supplement-2.pdf]

bioRxiv preprint doi: https://doi.org/10.64898/2026.05.20.726153; this version posted May 23, 2026. The copyright holder for this preprint (which was not certified by peer review) is the author/funder, who has granted bioRxiv a license to display the preprint in perpetuity. It is made available under a CC-BY 4.0 International license.

SUPPLEMENTARY MATERIALS

SUPPLEMENTARY METHODS

Fast empirical Bayes estimator

For sample i, let $\mathbf{e}_i$ denote the vector of residualized Y-gene expression values after covariate adjustment and centering relative to the reference samples. Under the empirical Bayes measurement model,

$$\mathbf{e}_i \mid \lambda_i, \boldsymbol{\Sigma} \sim N(-\lambda_i \mathbf{1}, \boldsymbol{\Sigma}),$$

where $\lambda_i \geq 0$ is the latent Y-expression depletion on the log2 scale, $\mathbf{1}$ is a vector of ones across the Y genes, and $\boldsymbol{\Sigma}$ is the residual covariance matrix estimated from the reference samples.

The generalized least-squares estimate of λ_i is

$$\hat{\lambda}_i = -\frac{\mathbf{1}^{\mathrm{T}} \boldsymbol{\Sigma}^{-1} \mathbf{e}_i}{\mathbf{1}^{\mathrm{T}} \boldsymbol{\Sigma}^{-1} \mathbf{1}},$$

with standard error:

$$\tau = (\mathbf{1}^{\mathrm{T}} \boldsymbol{\Sigma}^{-1} \mathbf{1})^{-1/2}.$$

Equivalently, after projecting the multigene residual vector into the one-dimensional depletion score, the likelihood is

$$\hat{\lambda}_i \mid \lambda_i \sim N(\lambda_i, \tau^2).$$

We used an exponential prior on nonnegative depletion,

$$\lambda_i \sim \mathrm{Exp}(r),$$

with default rate $r = 20$.

Therefore, the posterior density was

$$p(\lambda_i \mid \hat{\lambda}_i, \tau) = \frac{\phi(\hat{\lambda}_i; \lambda_i, \tau^2)\, r e^{-r\lambda_i}\, I(\lambda_i \geq 0)}{\int_0^\infty \phi(\hat{\lambda}_i; u, \tau^2)\, r e^{-ru}\, du},$$

bioRxiv preprint doi: https://doi.org/10.64898/2026.05.20.726153; this version posted May 23, 2026. The copyright holder for this preprint (which was not certified by peer review) is the author/funder, who has granted bioRxiv a license to display the preprint in perpetuity. It is made available under a CC-BY 4.0 International license.

where $\phi(\cdot;\mu,\sigma^2)$ denotes the normal density.

In implementation, this posterior was evaluated on a uniform grid of λ_i values from 0 to 2. For grid point λ_k, the normalized posterior weight was

$$w_{ik} = \frac{\exp\left[-\frac{1}{2}\left(\frac{\hat{\lambda}_i-\lambda_k}{\tau}\right)^2 - r\lambda_k\right]}{\sum_\ell \exp\left[-\frac{1}{2}\left(\frac{\hat{\lambda}_i-\lambda_\ell}{\tau}\right)^2 - r\lambda_\ell\right]}.$$

Posterior means, medians, and 95% credible intervals were computed from these grid weights. The depletion parameter was transformed to the cellular LOY fraction by

$$f_i = 1 - 2^{-\lambda_i}.$$

bioRxiv preprint doi: https://doi.org/10.64898/2026.05.20.726153; this version posted May 23, 2026. The copyright holder for this preprint (which was not certified by peer review) is the author/funder, who has granted bioRxiv a license to display the preprint in perpetuity. It is made available under a CC-BY 4.0 International license.

SUPPLEMENTARY FIGURES

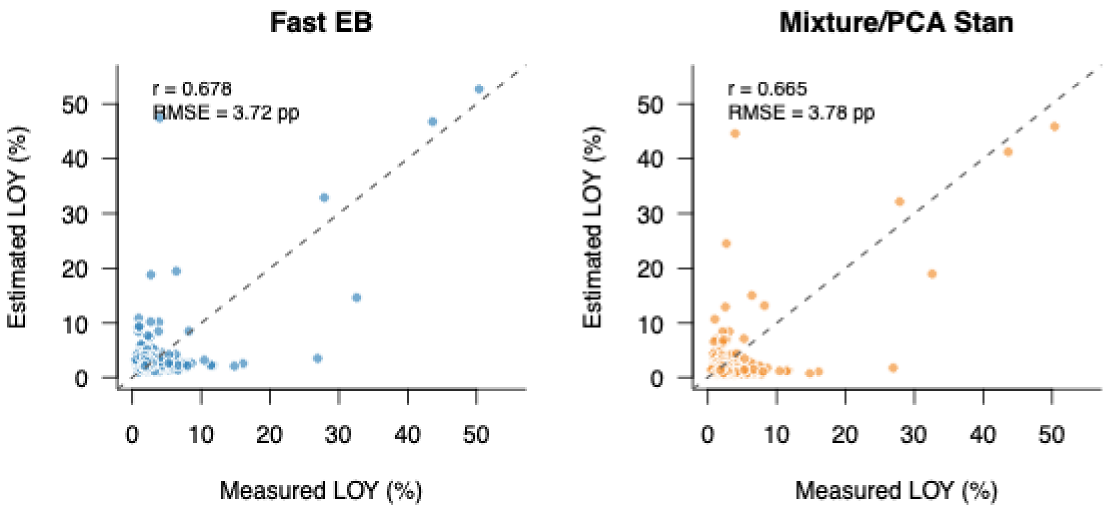

Supplementary Figure S1. Observed vs estimated LOY for main model variants. Scatterplots compare measured LOY with RNA-estimated LOY for the fast empirical Bayes estimator and mixture/PCA Stan model. Measured LOY was used only for validation.

bioRxiv preprint doi: https://doi.org/10.64898/2026.05.20.726153; this version posted May 23, 2026. The copyright holder for this preprint (which was not certified by peer review) is the author/funder, who has granted bioRxiv a license to display the preprint in perpetuity. It is made available under a CC-BY 4.0 International license.

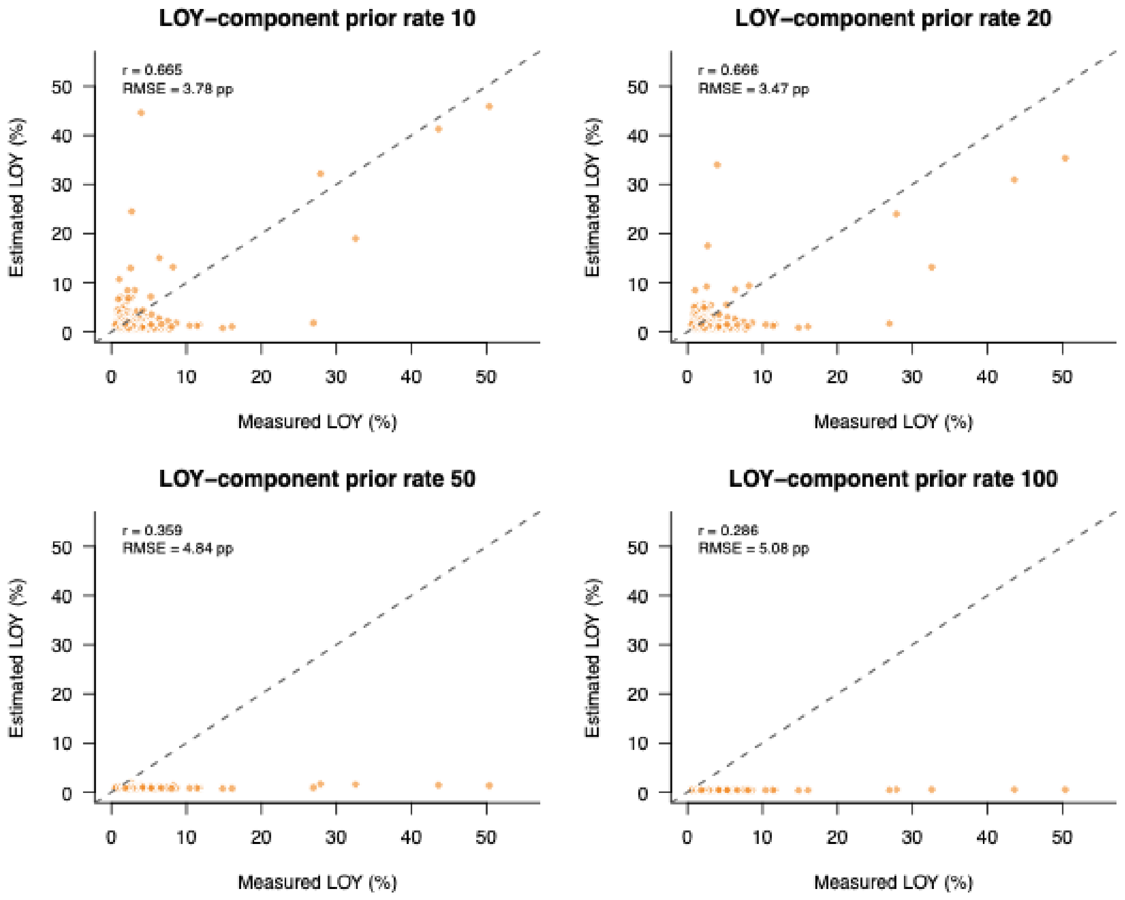

Supplementary Figure S2. Mixture/PCA Stan prior-sensitivity scatterplots. Observed vs estimated LOY scatterplots for mixture/PCA Stan models fit under LOY-component prior rates 10, 20, 50, and 100. Stronger priors shrink estimates toward zero and reduce recovery of high-LOY samples.

bioRxiv preprint doi: https://doi.org/10.64898/2026.05.20.726153; this version posted May 23, 2026. The copyright holder for this preprint (which was not certified by peer review) is the author/funder, who has granted bioRxiv a license to display the preprint in perpetuity. It is made available under a CC-BY 4.0 International license.

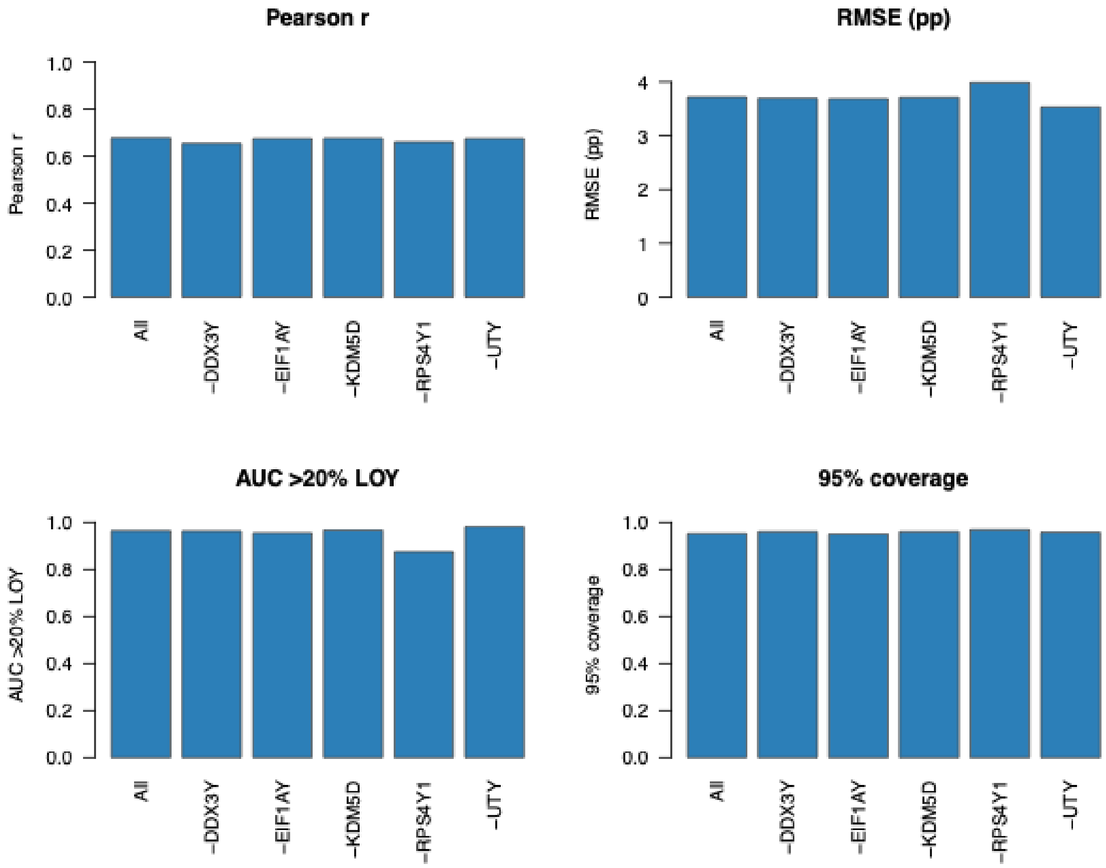

Supplementary Figure S3. Leave-one-Y-gene-out detailed metrics. Fast empirical Bayes validation metrics after dropping each Y gene in turn. Performance remains stable across leave-one-gene-out analyses, indicating that the signal is not driven by a single Y transcript.

bioRxiv preprint doi: https://doi.org/10.64898/2026.05.20.726153; this version posted May 23, 2026. The copyright holder for this preprint (which was not certified by peer review) is the author/funder, who has granted bioRxiv a license to display the preprint in perpetuity. It is made available under a CC-BY 4.0 International license.

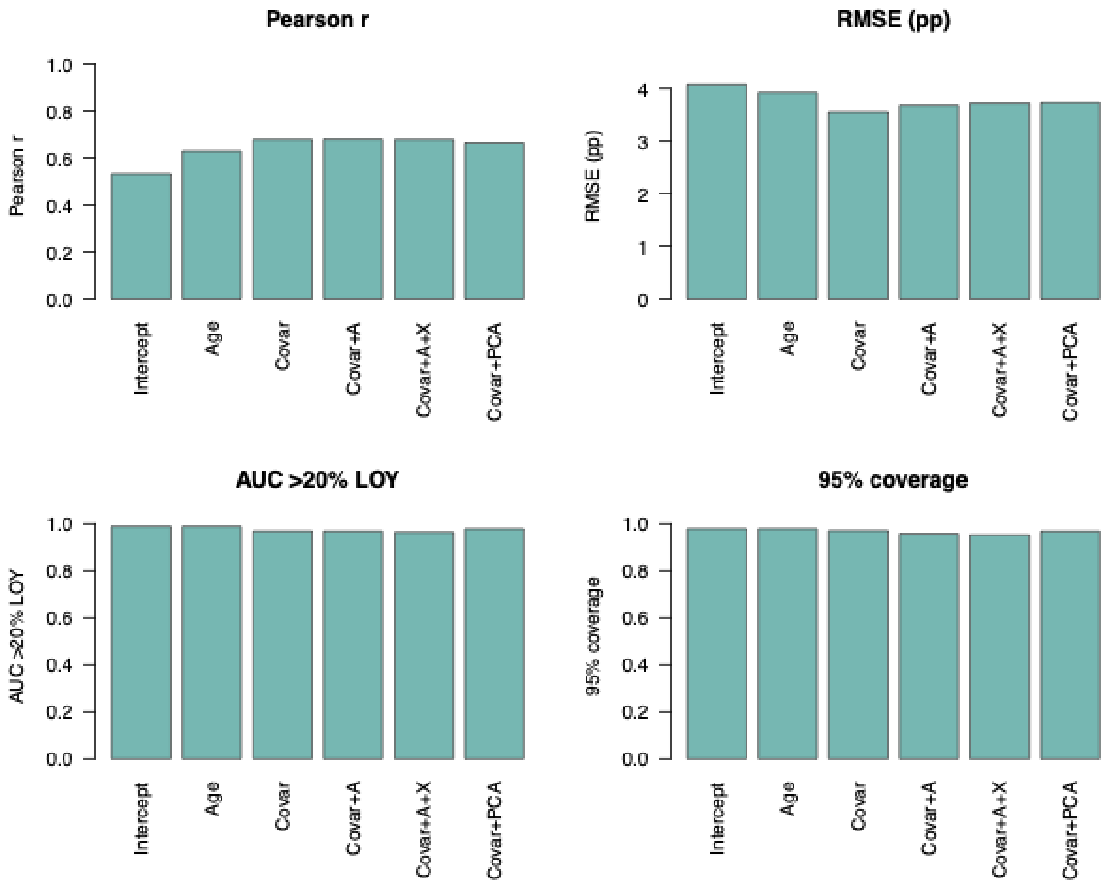

Supplementary Figure S4. Control-gene/covariate ablation detailed metrics. Validation metrics for empirical Bayes models fit with progressively richer covariate and control-gene adjustment sets.

bioRxiv preprint doi: https://doi.org/10.64898/2026.05.20.726153; this version posted May 23, 2026. The copyright holder for this preprint (which was not certified by peer review) is the author/funder, who has granted bioRxiv a license to display the preprint in perpetuity. It is made available under a CC-BY 4.0 International license.

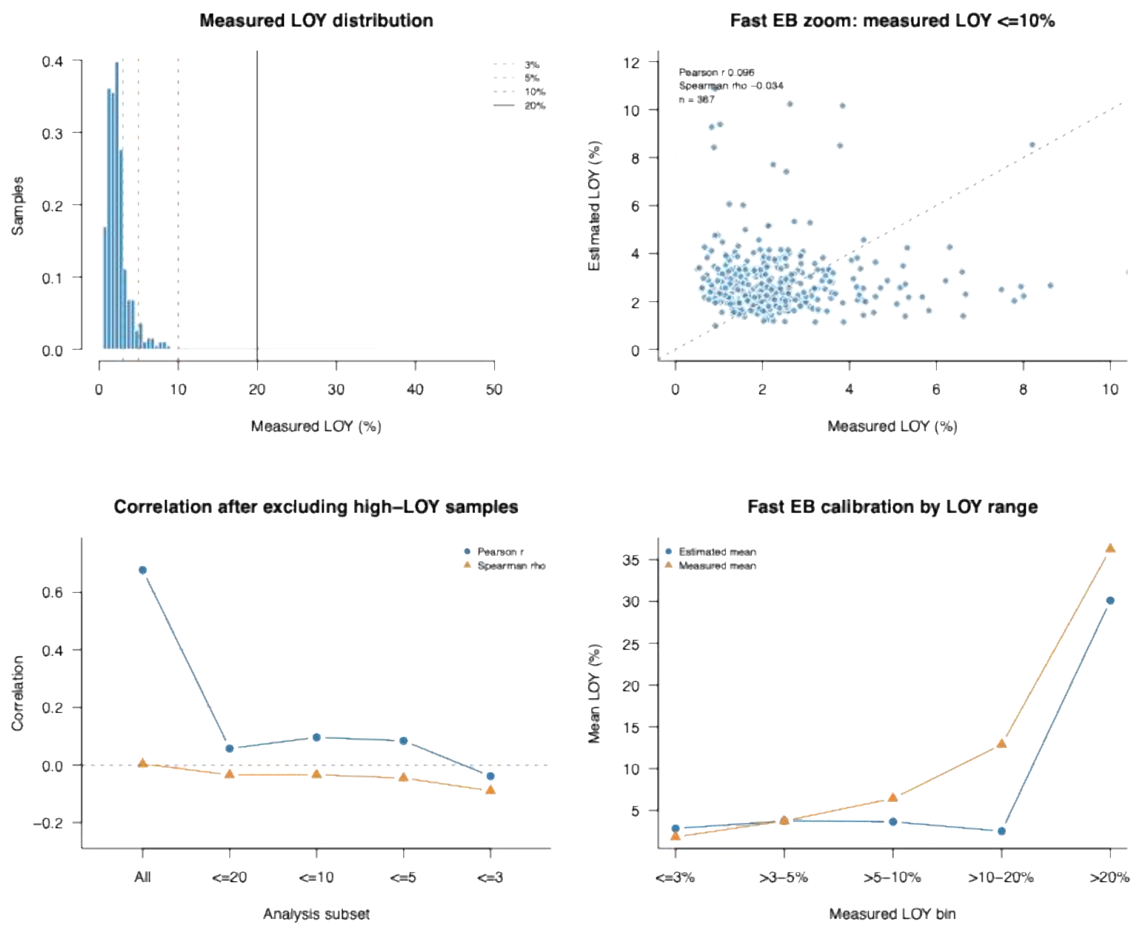

Supplementary Figure S5. Low-LOY and high-outlier diagnostics. Distribution of measured LOY in the GTEx validation samples, Fast EB scatterplot restricted to measured LOY ≤10%, correlations after excluding high-LOY samples, and mean observed versus estimated LOY by measured LOY range. These diagnostics show that overall Pearson correlation is driven mainly by the small number of high-LOY samples, whereas monotonic ranking within the low-LOY range is limited.

bioRxiv preprint doi: https://doi.org/10.64898/2026.05.20.726153; this version posted May 23, 2026. The copyright holder for this preprint (which was not certified by peer review) is the author/funder, who has granted bioRxiv a license to display the preprint in perpetuity. It is made available under a CC-BY 4.0 International license.

SUPPLEMENTARY TABLES

Supplementary Tables.xlsx includes the following supplementary tables:

- **Supplementary Table S1. Full bootstrap metric table.**
- **Supplementary Table S2. Hierarchical Bayesian posterior summaries.**
- **Supplementary Table S3a. GSE279480 sample manifest.**
- **Supplementary Table S3b. GSE279480 preprocessing summary.**
- **Supplementary Table S4. Gene-level stimulation effects.**
- **Supplementary Table S5. Low LOY and outlier sensitivity metrics.**
- **Supplementary Table S6. LOY range stratified calibration.**
